# Emerging paradigm: Molecularly targeted therapy with Dabrafenib and Trametinib in recurring pediatric gliomas with BRAF mutations: A narrative review

**DOI:** 10.1097/MD.0000000000040735

**Published:** 2024-12-06

**Authors:** Maryam Abdul Wahid, Muhammad Taimur Khan, Jawairya Muhammad Hussain, Hurais Malik, Shahood Ahmed Umar, Sanila Mughal, Muhammad Hasanain, Muhammad Umair Anjum, Mohammed Mahmmoud Fadelallah Eljack

**Affiliations:** a Dow Medical College Karachi, Karachi, Pakistan; b Abbottabad International Medical College, Abbottabad, Pakistan; c Karachi Medical and Dental College, Karachi, Pakistan; d Fazaia Ruth Pfau Medical College, Karachi, Pakistan; e Ziauddin Medical College, Karachi, Pakistan; f Dow Medical College, Karachi, Pakistan; g Aga Khan University Hospital, Internal Medicine, Karachi, Pakistan; h Dow Medical College, Dow University of Health Sciences, Karachi, Pakistan; i Faculty of Medicine and Health Sciences, University of Bakht Alruda, Ad Duwaym, Sudan; j Kassala Teaching Hospital, Kassala, Sudan.

**Keywords:** BRAF mutation, combination therapy, Dabrafenib, Gliomas, pediatrics, trametinib

## Abstract

Gliomas are tumors arising in the central nervous system, frequently associated with Class I mutations and BRAF fusions. These mutations are adverse prognostic factors in juvenile gliomas, leading to high rates of recurrence and poor response to current treatments. The blood-brain barrier and the heterogeneity of gliomas complicate the development of a single treatment strategy for all cases. This review aims to evaluate the efficacy and safety of combination therapies, particularly Dabrafenib and Trametinib, in pediatric gliomas with BRAF V600 mutations and discusses their potential in improving clinical outcomes. A review of recent clinical trials was conducted to assess the impact of targeted therapies, especially the combination of Dabrafenib and Trametinib, on glioma treatment outcomes. Additional therapies are also explored. Combination therapy with Dabrafenib, a BRAF kinase inhibitor, and Trametinib, a MEK inhibitor, has shown significant improvement in overall survival and progression-free survival for pediatric patients with BRAF V600-mutant gliomas. Recent clinical data from 2023 demonstrated enhanced tumor control, reduced relapse rates, and improved safety profiles compared to conventional therapies. Dabrafenib and Trametinib offer a promising targeted therapy for juvenile gliomas with BRAF V600 mutations, with better survival outcomes and manageable safety profiles. However, challenges remain in managing side effects such as fever, headache, lethargy, and rash. Further research into resistance mechanisms and long-term effects is necessary to optimize treatment strategies. Other therapies, such as everolimus and Selumetinib, also show potential and warrant further investigation.

## 1. Introduction

Gliomas are tumors that originate in the Central Nervous System, initially resembling healthy brain glial cells, hence the name. Pediatric gliomas, particularly those with V-Raf Murine Sarcoma Viral Oncogene Homolog B (BRAF) mutations, present significant challenges in neuro-oncology with an incidence rate of 3 to 4 cases per 100,000 children.^[1]^ Historically, children with BRAF V600E-mutated gliomas have had poor outcomes with conventional chemoradiotherapy strategies. These tumors are associated with shorter response durations and worse long-term outcomes compared to non-BRAF V600E gliomas.^[2]^ The BRAF V600E mutations is an unfavorable prognostic factor in childhood gliomas leading to high rates of tumor recurrence and poor response to standard treatments.^[3]^ Furthermore, treating pediatric gliomas presents several limitations. First, the blood-brain barrier restricts the delivery of drugs to the brain, making it difficult to achieve effective drug concentrations. Second, surgery may not always be feasible due to tumor location or size. Additionally, chemotherapy and radiation can cause long-term side effects in developing brains. Finally, the heterogeneity of gliomas complicates the search for a single treatment approach that works for all cases.^[4]^

Despite these challenges, the emergence of targeted therapies has opened new alternatives to treat these tumors. The landscape of cancer therapy is rapidly evolving, with novel approaches such as nanotechnology-based drug delivery systems gaining prominence. Studies exploring nanovesicles and nanoliposomes for the targeted delivery of cancer therapeutics, such as silymarin in cancer therapy and doxorubicin in osteosarcoma, represent significant strides towards improving the precision and efficacy of treatments.^[5]^ These advancements, though primarily focused on different cancer types, underscore the broader trend towards personalized and targeted approaches in oncology. Similarly, nanomaterials are being investigated for the diagnosis and treatment of cancers such as those in the head and neck, further illustrating the role of innovative delivery systems in modern cancer care.^[6]^ In parallel, molecularly targeted therapies like the BRAF and mitogen-activated extracellular kinase (MEK) inhibitors Dabrafenib and Trametinib, respectively are emerging as promising treatments for pediatric gliomas harboring BRAF mutations, providing a targeted approach with potential to improve patient outcomes in this challenging disease context.^[7,8]^ Combination therapy has shown better clinical responses and lower toxicity than chemotherapy in children with BRAF V600E mutations and low-grade gliomas.^[9]^ This led to a major paradigm shift in the treatment of these patients.^[9]^This underscores the broader shift towards targeted and personalized therapies.

The combination of Dabrafenib and Trametinib offers a targeted treatment specifically for pediatric gliomas with BRAF V600 mutations. It has demonstrated higher response rates and longer progression-free survival compared to conventional chemotherapy. Additionally, it has a more favorable safety profile, with fewer serious adverse events.^[10]^ This FDA-approved combination, also available in liquid form, represents a significant advancement in the treatment of pediatric gliomas. The rationale for this narrative review is to provide a comprehensive synthesis of the current evidence regarding the use of these targeted therapies in pediatric gliomas, focusing on their mechanisms of action, clinical efficacy, safety profiles, and the potential for integrating precision medicine and pharmacogenomics into treatment approaches.

A key research gap identified in the existing literature is the limited understanding of long-term outcomes and potential resistance mechanisms associated with prolonged Dabrafenib and Trametinib therapy in pediatric patients. While initial studies have shown favorable response rates and manageable toxicity profiles, there is a lack of data on the durability of these responses and the optimal strategies to overcome or prevent resistance. Additionally, the integration of pharmacogenomic data to tailor treatment regimens remains underexplored, underscoring the need for further research to optimize personalized treatment approaches. This review aims to address these gaps by analyzing recent clinical trials, evaluating the implications of resistance mechanisms, and exploring emerging therapies and future directions in the management of pediatric gliomas with BRAF mutations.

## 2. Materials and methods

A comprehensive literature search was conducted to identify relevant studies and articles on the use of Dabrafenib and Trametinib in the treatment of pediatric BRAF-mutant gliomas. The databases searched included PubMed, Embass, Scopus, Web of Science, and Google Scholar. The search terms used were a combination of keywords and MeSH terms such as “Dabrafenib,” “Trametinib,” “BRAF-mutant glioma,” “pediatric glioma,” “BRAF V600E mutation,” “targeted therapy,” and “combination therapy,” the detail search string is available in Supplementary digital content. http://links.lww.com/MD/O66 The search was limited to articles published in English from January 2010 to June 2024.

Eligibility criteria for the review included original research articles, clinical trials, and case reports that focused on the use of Dabrafenib and Trametinib in pediatric patients with BRAF-mutant gliomas. Studies were required to report on the efficacy, safety, and clinical outcomes of these treatments. Additionally, reviews and meta-analyses that provided comprehensive insights into the mechanisms of action, clinical application, and potential benefits of Dabrafenib and Trametinib in pediatric BRAF-mutant gliomas were included. Only articles published in English from January 2010 to June 2024 were considered. Studies focusing solely on adult patients, preclinical studies without direct clinical relevance, abstracts, conference proceedings, and editorials without sufficient data were excluded from the review.

Following the search, we used EndNote Reference Library software to identify and remove duplicate articles. Two authors (M.T.K. and M.A.W.) independently screened the titles and abstracts, to assess eligibility.

## 3. Results

### 3.1. BRAF mutant gliomas: an overview

Pediatric gliomas and low-grade gliomas (pLGGs) account for approximately 30% of pediatric central nervous system tumors, consisting heterogeneous groups of tumors with primary glial or mixed glial histology.^[4]^ The BRAF V600E mutation is the most prevalent BRAF alteration in gliomas, particularly in juvenile low-grade astrocytoma, pleomorphic astrocytoma, gangliogliomas, and epithelioid glioblastoma.^[11]^ These genetic changes have facilitated the development of targeted therapies, which have improved the overall prognosis of various solid tumors by reducing recurrence and progression rates.^[12]^

BRAF-mutant pediatric glioma is a key subtype of low-grade glioma in children.^[13]^ These tumors often involve Class I mutations and subsequently develop BRAF fusions, mostly seen in pilocytic astrocytoma and pLGG histologies. They are usually cystic and histologically characterized by dense cellularity, nuclear atypia, pleomorphism and multinucleation. The occurrence of BRAF V600E mutations in childhood gliomas is about 23%, and around 10% in WHO stages III and IV.^[14]^ This mutation is more common in low-grade tumors and is associated with poor responses to chemotherapy.^[7]^ It has a higher incidence in epithelioid glioblastoma, anaplastic polymorphic cholangiocarcinoma, ganglioglioma, and anaplastic ganglioglioma.^[7]^

### 3.2. Dabrafenib and Trametinib: mechanism of action

#### 3.2.1. Dabrafenib

Dabrafenib is a reversible, selective inhibitor of ATP and RAF kinases, including mutant BRAF. In pediatric patients, dabrafenib specifically targets abnormal BRAF proteins, particularly the BRAF V600E mutation, which is common in pediatric low-grade gliomas. By inhibiting mutant BRAF, dabrafenib disrupts the MAPK/ERK signaling pathway, leading to reduced cell proliferation and potential apoptosis of tumor cells. Pediatric gliomas often harbor BRAF fusions (e.g., KIAA1549-BRAF) that also respond to dabrafenib, though the drug’s efficacy may vary depending on the specific mutation type and tumor microenvironment. Dabrafenib has a higher affinity for BRAF mutations, including BRAF V600K, BRAF V600E, and BRAF V600D, making it particularly effective in targeting these mutations in pediatric tumors.^[15]^

#### 3.2.2. Trametinib

Trametinib is a reversible and selective inhibitor of the activation and kinase activity of mitogen-activated extracellular kinase (MEK) 1 and 2. In pediatric patients, Trametinib’s inhibition of MEK1 and MEK2 prevents the activation of the downstream ERK pathway, which is crucial for cell proliferation and survival. By blocking this pathway, trametinib effectively inhibits the growth of BRAF mutant cells. This mechanism is particularly beneficial in pediatric gliomas where the MAPK/ERK pathway is often hyperactivated due to BRAF mutations. Trametinib’s role in targeting these signaling proteins makes it an essential part of combination therapy with dabrafenib, especially in managing low-grade gliomas in children.^[16,17]^

#### 3.2.3. Synergistic action

Dabrafenib and trametinib work synergistically by targeting different components of the same signaling pathway, enhancing their overall efficacy. Dabrafenib inhibits the upstream BRAF kinase, while trametinib inhibits the downstream MEK proteins in the MAPK/ERK pathway. This dual blockade results in a more complete inhibition of the signaling cascade, reducing the likelihood of resistance development and leading to more effective tumor control.^[18]^

## 4. Discussion

### 4.1. Recent clinical trials and studies

Recent clinical trials have established the efficacy and safety of BRAF-targeted therapies for treating pediatric gliomas with BRAF mutations. On June 22, 2022, the FDA granted accelerated approval for the combination of dabrafenib and trametinib for the treatment of BRAF V600E-mutated unresectable or metastatic solid tumors in both adults and pediatric patients over six years of age.^[19]^

#### 4.1.1. Hargrave et al (2023)^[19]^

*Trial Design*: Enrolled 41 patients with BRAF-mutated pediatric high-grade gliomas (pHGG) across 28 sites in 13 countries from December 2017 to August 2020. The median age was 13 years, and the median time since diagnosis was 17.4 months. Glioblastoma multiforme was the most common histological type.

*Efficacy*: The combination therapy achieved a 56.1% overall response rate (ORR), including 12 complete responses (CRs) and 11 partial responses (PRs). Approximately 90% of patients experienced a 50% reduction in tumor size, with 50% achieving a 100% reduction. The median duration of response (DOR) was 22.2 months, and median progression-free survival (PFS) was 9.0 months.

*Safety*: All patients experienced adverse events (AEs), with 68.3% having grade ≥ 3 AEs. The most common AEs were pyrexia and headache. Serious AEs were reported in 61% of patients, with three fatalities not related to treatment. Median overall survival (OS) was 32.8 months, with 12- and 24-month OS rates of 76.3% and 58.6%, respectively.

#### 4.1.2. Bouffet et al (2023)^[20]^

*Trial Design*: This study compared dabrafenib plus trametinib to chemotherapy in 110 patients with pediatric gliomas from September 2018 to December 2020. The median follow-up was 18.9 months.

*Efficacy*: Dabrafenib plus trametinib resulted in a significantly higher ORR of 47% compared to 11% with chemotherapy. Clinical benefit (complete or partial response or stable disease for ≥ 24 weeks) was 86% in the dabrafenib plus trametinib group versus 46% in the chemotherapy group. Median PFS was 20.1 months with the combination versus 7.4 months with chemotherapy.

*Safety*: Fewer grade ≥ 3 AEs were observed with dabrafenib plus trametinib (47%) compared to chemotherapy (94%). Common AEs included pyrexia and headache. There were no deaths in the dabrafenib plus trametinib group, while one death occurred in the chemotherapy group. The combination therapy showed better global health and fatigue scores.

#### 4.1.3. Bouffet et al (2022)^[21]^

*Trial design*: A phase I/II study assessed trametinib alone and in combination with dabrafenib in 139 patients with BRAF V600E-mutant low-grade gliomas (LGG) from January 2015 to December 2020. The median exposure was 24 months for trametinib monotherapy and 21 to 24 months for the combination.

*Efficacy*: Trametinib monotherapy showed a 15% objective response rate and 46% stable disease rate. The combination therapy yielded a 25% response rate and 64% stable disease, with a median DOR of 33.6 months. Median PFS was 16.4 months for trametinib alone and 36.9 months for the combination.

*Safety*: Common AEs included paronychia and dry skin for trametinib, pyrexia and dry skin for the combination. Dose adjustments were frequent, but no on-treatment deaths occurred.

These studies collectively demonstrate that dabrafenib and trametinib represent a highly effective therapeutic option for BRAF V600E-mutant pediatric gliomas. The combination therapy shows superior efficacy in terms of ORR, PFS, and DOR compared to traditional chemotherapy. A comprehensive overview of the progression-free survival and overall survival outcomes is presented in Table 1. Safety profiles suggest manageable AEs, with no on-treatment deaths reported in recent trials. These AEs were found frequently leading to dose reduction, interruption, and discontinuation of Dabrafenib plus Trametinib; however, none of the adverse effects were fatal. An overview of the major side effects and their impact on treatment reduction, interruption, and discontinuation from recent clinical trials is summarized in Table 2.

**Table 1:** Major adverse events and their impact on treatment adjustment

Title	ClinicalTrials.gov registration number	Adverse events		AEs leading to			Death due TRAEs
				Dose reduction	Dose interruption	Discontinued treatment	
Efficacy and Safety of Trametinib Monotherapy or in Combination with Dabrafenib in Pediatric BRAF V600-Mutant Low-Grade Glioma	NCT02124772	trametinib monotherapy group (n = 13)	most common TRAEs were paronychia, diarrhea, and dry skin.	9 (69%) patients	10 (77%) patients	7 (54%) patients	None were treatment related.
		combination therapy group (n = 36)	most common TRAEs were pyrexia and dry skin.	11 (31%) patients	26 (72%) patients	8 (22%) patients	None were treatment related.
Dabrafenib plus Trametinib in Pediatric Glioma with BRAF V600 Mutations	NCT02684058.	47% of the patients receiving dabrafenib + trametinib and 94% of those receiving chemotherapy reported grade 3 or higher AEs.		45 (62%) receiving dabrafenib, 14 (19%) receiving trametinib, 21 (64%) receiving carboplatin, and 11 (33%) receiving vincristine	56 (77%) receiving dabrafenib, 53 (73%) receiving trametinib, 23 (70%) receiving carboplatin, and 22 (67%) receiving vincristine	12 patients assigned to receive dabrafenib plus trametinib; 16 in the chemotherapy group.	None among the patients who received dabrafenib plus trametinib; 1 (from low-grade glioma) in the chemotherapy group.
Phase II Trial of Dabrafenib Plus Trametinib in Relapsed/Refractory BRAF V600-Mutant Pediatric High-Grade Glioma	NCT02684058.	All patients experienced at least one AE, with 28 patients (68.3%) experiencing an AE of grade ≥ 3. The most common AEs were pyrexia, headache, dry skin, vomiting and diarrhea.		26 (63.4%) patients		2 patients (5%) (both had rash).	None were treatment related.

AEs = adverse events, TRAEs = treatment-related adverse events.

**Table 2:** Efficacy of Dabrafenib and Trametinib combined therapy

Title	First Author	ClinicalTrials.gov registration number	Study design	Patient populations	Efficacy		
					Primary end point ORR (CR + PR)	Median PFS	Median DOR
Efficacy and Safety of Trametinib Monotherapy or in Combination with Dabrafenib in Pediatric BRAF V600-Mutant Low-Grade Glioma	Eric Bouffet	NCT02124772	Four-part, phase I/II study	139 patients; 91 received trametinib monotherapy and 48 received dabrafenib + trametinib.	15% (95% CI, 1.9–45.4) for monotherapy (n = 13) and 25% (95% CI, 12.1–42.2) for combination (n = 36).	16.4 months (95% CI, 3.2 to NR) for trametinib monotherapy (n = 13) and 36.9 months (95% CI, 36.0 to NR) in the combination therapy (n = 36)	NR in the trametinib monotherapy and 33.6 months (95% CI, 11.2 to NR), in the combination therapy.
Dabrafenib plus Trametinib in Pediatric Glioma with BRAF V600 Mutations	Eric Bouffet	NCT02684058	phase II trial.	110 patients; 73 received dabrafenib + trametinib and 37 received standard chemotherapy.	47% for dabrafenib plus trametinib (n = 34) and 11% for chemotherapy (n = 4).	20.1 months (95% CI, 12.8 to not evaluable) for dabrafenib plus trametinib and 7.4 months (95% CI, 3.6–11.8; *P* < .001) for chemotherapy	20.3 (12.0–NE) for dabrafenib plus trametinib and NE for chemotherapy
Phase II Trial of Dabrafenib Plus Trametinib in Relapsed/Refractory BRAF V600-Mutant Pediatric High-Grade Glioma	Darren R Hargrave	NCT02684058.	Phase II study.	41 patients of BRAF V600-mutant HGG.	56.1% (95% CI, 39.7–71.5). 12 confirmed CRs (29.3%) and 11 PRs (26.8%).	9.0 months (95% CI, 5.3–24.0 months)	22.2 months (95% CI, 7.6 months to not reached [NR]).

CR = complete response, DOR = duration of response, HGG = high-grade glioma, LGG = low-grade glioma, NE = not evaluable, NR = not reached, ORR = overall response rate, PFS = progression-free survival, PR = partial response.

### 4.2. Major side effects and tolerability

The combination of dabrafenib (a BRAF inhibitor) and trametinib (a MEK inhibitor) has been studied extensively in various cancers, including pediatric gliomas with BRAF mutations. The Children undergoing combination therapy may experience several side effects. Fever is a common issue, often managed with fever-reducing drugs. Skin-related side effects, such as thickening of the skin, acne-like rashes, dry skin, and skin rashes are also possible. Gastrointestinal problems including nausea, vomiting, diarrhea, and stomach pain, can occur as well. Fatigue, which may lead to extreme tiredness or weakness, is another potential side effect. Notably, trametinib and dabrafenib may affect the heart’s electrical system or rhythm, necessitating monitoring with electrocardiograms (ECGs). Additionally, some pediatric patients have experienced eye-related side effects such as uveitis and retinal vein blockage.^[10,20–22]^ Overall, while the combination of dabrafenib and trametinib shows a demonstrates a manageable safety profile and good tolerability in treating pediatric gliomas with BRAF mutations, vigilant monitoring and proactive management of adverse events (AEs) are crucial to ensuring optimal outcomes for young patients.

### 4.3. Dosages

In pediatric populations, particularly those with gliomas harboring BRAF mutations, the safety and tolerability of combination therapy must be carefully considered due to differences in drug metabolism and side effect profiles compared to adults. Studies have shown promising efficacy with an acceptable safety profile in pediatric patients, but close monitoring is essential to manage AEs effectively. Pediatric dosing may require adjustments based on body surface area and the patient’s overall health status to minimize AEs while maintaining therapeutic efficacy.

The dose of dabrafenib and trametinib depends on the patient’s body weight. For individuals weighing 26 to 37 kg, the recommended dose is 75 mg for dabrafenib and 1 mg of trametinib. Those weighing 38 to 50 kg should take 100 mg of dabrafenib and 1.5 mg of trametinib, while those weighing 51 kg or more should take 150 mg of dabrafenib and 2 mg of trametinib. Dabrafenib is to be taken orally-twice a day, while trametinib should be taken once daily.^[23,24]^

### 4.4. Precision medicine and pharmacogenomics

The integration of precision medicine and pharmacogenomics into treatment paradigms has shown significant promise in enhancing clinical outcomes. In pediatric oncology, precision medicine involves tailoring treatment strategies to the individual genetic profiles of patients. Pharmacogenomic profiling enables the identification of patients most likely to benefit from targeted therapies, ensuring more precise and effective interventions.^[25]^ The presence of BRAF V600E mutations serves as a critical biomarker for selecting appropriate targeted treatments, optimizing therapeutic outcomes while minimizing adverse effects.^[20,26,27]^ Comprehensive molecular profiling of gliomas can further identify additional genetic alterations that might influence therapeutic response. For instance, concurrent mutations in genes involved in the PI3K/AKT pathway may suggest potential resistance to BRAF/MEK inhibition, indicating the need for combination therapies that target multiple pathways.^[28]^ Additionally, specific gene expression signatures associated with MAPK pathway activation can serve as predictive biomarkers, with high expression of pathway components correlating with better therapeutic outcomes.^[29]^

Histological subtypes and anatomical locations of gliomas also play a role in predicting treatment response, with low-grade gliomas harboring BRAF V600E mutations showing more favorable outcomes compared to high-grade variants.^[30]^ Furthermore, the loss of the tumor suppressor PTEN can activate the PI3K/AKT pathway, potentially leading to resistance against BRAF and MEK inhibitors. Thus, assessing PTEN status may help identify patients who might benefit from additional pathway inhibitors.^[20]^

Integrating pharmacogenomics into clinical practice necessitates routine genetic testing and the adoption of personalized treatment protocols based on pharmacogenomic data. This approach not only improves the precision of therapeutic interventions but also facilitates continuous monitoring and adaptation of treatment plans, ensuring sustained disease control and the management of potential resistance mechanisms. However, the successful implementation of precision medicine in pediatric gliomas with BRAF mutations requires addressing ethical and economic considerations, such as equitable access to genetic testing and targeted therapies, and evaluating the cost-effectiveness of these advanced treatment modalities. By focusing on these aspects, the integration of precision medicine and pharmacogenomics into treatment paradigms for pediatric gliomas with BRAF mutations can significantly transform clinical practice and improve patient outcomes.

### 4.5. Resistance

Following initial responses to BRAF/MEK inhibition, some patients will ultimately develop secondary resistance, driven by alterations that can be either dependent on or independent of the MAPK pathway. For instance, Neuroblastoma RAS mutations can activate the MAPK pathway through CRAF dimerization, leading to resistance. Similarly, non-BRAF V600 alterations, such as BRAF fusions, can evade targeted inhibition. Changes in the MEK, ERK, and COT pathways also contribute to resistance. Additionally, resistance may arise from the upregulation of receptor tyrosine kinases or activation of the PI3K/AKT pathway.^[31,32]^

#### 4.5.1. Challenges and limitations

Numerous studies examining the use of dabrafenib and trametinib in pediatric gliomas suffer from small sample sizes and incomplete data, making it difficult to definitively determine their effectiveness and safety. Comparative analyses between this combined therapy and alternative treatments, such as various chemotherapy regimens and other targeted therapies, are notably lacking. This gap complicates efforts to fully understand the relative advantages and disadvantages of each approach. The long-term efficacy and safety profiles of dabrafenib and trametinib in pediatric populations are insufficiently documented. Further investigation is needed to evaluate potential delayed adverse effects and overall survival rates over extended periods.

Although the mechanisms of action for dabrafenib and trametinib are generally well understood, detailed insights into the molecular pathways involved, particularly in regard to resistance mechanisms in pediatric gliomas, remain limited. Despite these therapies exhibiting lower toxicity compared to conventional chemotherapy, managing common adverse reactions such as fever, headache, fatigue, and rash continues to be challenging. More research is needed to optimize supportive care strategies.

Current research primarily focuses on specific subgroups of pediatric patients. As a result, understanding how diverse genetic backgrounds and tumor characteristics influence treatment outcomes is essential for developing personalized therapeutic approaches.

#### 4.5.2. Emerging therapies and future directions

Recent developments in molecularly targeted therapies for pediatric gliomas with BRAF mutations have highlighted several promising avenues. In pediatric low-grade gliomas (PLGAs), sorafenib has paradoxically accelerated tumor growth regardless of BRAF or NF1 status, underscoring the complexity of these tumors.^[33]^ In contrast, everolimus has shown promise as a well-tolerated alternative for radiographically progressive and recurrent pediatric low-grade gliomas (LGGs) when administered orally on a daily basis.^[34]^ Selumetinib, another notable MEK inhibitor, has demonstrated efficacy in extending disease stability in children with progressive LGGs harboring BRAF or NF1 mutations.^[35]^

The recommended pediatric phase 2 dose (RP2D) for cobimetinib, at 0.8 mg/kg for tablets and 1.0 mg/kg for suspension, has been well tolerated among pediatric and young adult patients with relapsed solid tumors^[36]^ Mirdametinib (PD-0325901) is currently under investigation in an open-label, multi-center trial (NCT04923126), with preclinical studies suggesting superior blood-brain barrier penetration compared to other MEK inhibitors.^[37]^

Ongoing Phase II trials for pediatric high-grade gliomas (pHGG) are exploring various agents, including nimotuzumab (NCT03620032, NCT04532229, NCT00561873, NCT00600054), erlotinib (NCT00418327), and cetuximab (NCT01884740), both as monotherapies and in combination with mTOR inhibitors. While high recurrence rates due to acquired tumor resistance present a challenge to Epidermal Growth Factor Receptor inhibition, combination therapies, particularly with PI3K inhibitors, have shown improved treatment responses in pHGG patients.^[38]^

Disparities in survival rates among childhood cancers highlight the urgent need for more effective treatment strategies. Integrating molecular profiling with a panel approach into standard testing protocols can identify key genetic drivers and tailor therapies accordingly.^[39]^ However, significant barriers remain, including the blood-brain barrier, tumor heterogeneity, immune-compromised tumor environments, and antigen escape phenomenon. Overcoming these obstacles is crucial for advancing immunotherapeutic strategies in pediatric cancer treatment^[40]^

### 4.6. Efficacy and safety of other combination therapies in pediatric gliomas

The MEK inhibitor cobimetinib has shown some benefits in treating pediatric low-grade gliomas (LGG), although the overall response rate (ORR) remains modest, with a partial response (PR) observed in only 5% of patients. Notably, some patients with neurofibromatosis type 1 (NF1)-associated LGG have experienced prolonged stable disease. However, treatment-related adverse events (TRAEs) led to the discontinuation of cobimetinib in six patients (11%): 6% during tablet dose escalation, 15% during suspension dosage increase, and 8% during expansion. There were 11 fatalities (20%) due to disease progression (PD) during the study. Investigator assessments have shown significant improvements in progression-free survival (PFS) and objective response rates (ORR) when cobimetinib is combined with the BRAF inhibitor vemurafenib.^[36]^

In BRAF mutant and vemurafenib-resistant cells, the combination therapy of romidepsin and interferon-alpha (IFN-α) effectively reduces melanoma invasiveness, induces mixed apoptotic and necroptotic cell death, and reverses resistance to BRAF inhibitors. This drug combination enhances the immune response by modulating IFN-γ levels, improving antigen presentation, and activating immune cells. It also inhibits immune checkpoints and downregulates critical oncogenes such as G2M checkpoint regulators, Myc, and E2F targets, which are essential for cancer progression.^[41]^

A Phase II study conducted by the Pediatric Brain Tumor Consortium (PBTC) evaluated the combination of bevacizumab and irinotecan in 35 children with recurrent pediatric low-grade glioma (pLGG), reporting a 2-year PFS of 47.8%. Confirmed responses were observed in 5.7% of the patients, with a median of 12 treatment cycles. Common toxicities in the trial included grade 1-2 hypertension, fatigue, epistaxis, and proteinuria, ranging from grades 1 to 4.^[42]^

In an ongoing Phase II clinical trial, PLGG-MEKTRIC (NCT05180825), the MEK inhibitor trametinib (MekinistTM) is being compared to standard chemotherapy with vinblastine over 18 courses of 4 weeks each in pediatric low-grade glioma and mixed glioneuronal tumors, including pleomorphic xanthoastrocytoma (PXA) without BRAF V600E mutation or NF1 correlation. The primary outcome measure is 3-year PFS, with additional data being collected on variations in PFS and overall survival (OS) based on molecular biomarkers.^[37]^

### 4.7. Further recommendations

Advancements in targeted therapy for pediatric oncology depend on the investigation of biomarkers and the incorporation of personalized medicine. While Trametinib and Dabrafenib have shown potential in treating BRAF-mutant gliomas, the development of reliable predictive biomarkers remains critical. Future research should focus on identifying and validating specific biomarkers that can accurately predict patient responses to Trametinib and Dabrafenib in pediatric BRAF-mutant gliomas. By elucidating the molecular markers associated with therapeutic outcomes, researchers can optimize treatment regimens, minimize adverse effects, and enhance the overall efficacy of these targeted therapies.

The identification of predictive biomarkers will significantly advance the management of pediatric BRAF-mutant gliomas, enabling the implementation of personalized medicine strategies that tailor interventions to individual patient profiles. This personalized approach has the potential to dramatically enhance treatment outcomes. Additionally, investigating the synergistic effects of combining these targeted therapies with other treatments, such as immunotherapy and advanced drug delivery systems capable of crossing the blood-brain barrier, holds considerable promise for improving therapeutic success. Such combinatorial strategies could lead to more effective and comprehensive treatment protocols, ultimately improving prognosis and quality of life for pediatric patients with BRAF-mutant gliomas.

## 5. Conclusion

In conclusion, the emerging paradigm of molecularly targeted therapies, particularly the combination of Dabrafenib and Trametinib, has revolutionized the treatment landscape for pediatric gliomas with BRAF mutations. These targeted therapies have demonstrated significantly improved clinical outcomes, including higher overall response rates, prolonged progression-free survival, and a better safety profile compared to conventional chemoradiotherapy. While the safety profile tends to be manageable, adverse events must be closely monitored and actively managed to improve patient outcomes. The fusion of precision medicine and pharmacogenomics marks a significant leap forward, enabling personalized therapy through gene and biomarker profile-based treatment optimization and toxicity reduction.

However, overcoming various resistance mechanisms remains a key challenge. Secondary resistance can arise from changes in the MAPK pathway, both dependent and independent. Monitoring molecular changes and having treatment plans that can be adjusted are essential. New MEK inhibitors and combination therapies have the potential to overcome resistance, but additional preclinical data are required to determine their relevance in clinical practice. Future developments in pediatric glioma therapy will rely on a comprehensive individualized approach, with molecular data used to inform treatment decisions and optimize processes. To improve outcomes, further research is needed into biomarkers, combination strategies, immunotherapies, and advanced drug delivery modalities.

The integration of targeted therapies within a precision medicine framework may transform how pediatric gliomas are treated. By applying molecular data, more individualized, effective, and sustainable approaches can be delivered to children, combining targeted medications with extensive supporting interventions. Advances in targeted therapy for pediatric cancer suggest that future studies should focus on overcoming resistance, developing new drugs and enhancing the supportive care to maximize the benefits of targeted therapy. This narrative review underscores the importance of continued research and clinical innovation to refine therapeutic strategies and ultimately improve the prognosis and quality of life for these young patients.

## Author contributions

**Conceptualization:** Maryam Abdul Wahid, Muhammad Taimur Khan.

**Supervision:** Muhammad Umair Anjum, Mohammed Mahmmoud Fadelallah Eljack.

**Visualization:** Jawairya Muhammad Hussain.

**Writing – original draft:** Maryam Abdul Wahid, Muhammad Taimur Khan, Jawairya Muhammad Hussain, Hurais Malik, Shahood Ahmed Umar.

**Writing – review & editing:** Hurais Malik, Sanila Mughal, Muhammad Hasanain, Muhammad Umair Anjum, Mohammed Mahmmoud Fadelallah Eljack.

## Supplementary Material


