# Supplementary material for: Emerging paradigm: Molecularly targeted therapy with Dabrafenib and Trametinib in recurring pediatric gliomas with BRAF mutations: A narrative review
Source: Medicine (Baltimore). 2024 Dec 6;103(49):e40735. doi: 10.1097/MD.0000000000040735 (PMC11630983; doi:10.1097/MD.0000000000040735)
Supplement: Supplementary file 1 [file medi-103-e40735-s001.docx]

**Supplementary Table: Search Strategy**

| **Databases** | **Search Strings** |
| --- | --- |
| MEDLINE | "dabrafenib," "GSK 2118436," "GSK2118436," "GSK-2118436," AND "trametinib," "JTP 74057," "JTP74057," "JTP-74057," "GSK 1120212," "GSK1120212," "GSK-1120212," AND "BRAF-mutant glioma," "pediatric glioma," "BRAF V600E mutation," "Glioma," "Gliomas," "Glial Cell Tumors," "Glial Cell Tumor," "Tumor, Glial Cell," "Tumors, Glial Cell," "Mixed Glioma," "Glioma, Mixed," "Gliomas, Mixed," "Mixed Gliomas," "Malignant Glioma," "Glioma, Malignant," "Gliomas, Malignant," "Malignant Gliomas," AND "BRAF" AND "targeted therapy," "combination therapy," "dual therapy." |
| Scopus | "dabrafenib," "GSK 2118436," "GSK2118436," "GSK-2118436," AND "trametinib," "JTP 74057," "JTP74057," "JTP-74057," "GSK 1120212," "GSK1120212," "GSK-1120212," AND "BRAF-mutant glioma," "pediatric glioma," "BRAF V600E mutation," "Glioma," "Gliomas," "Glial Cell Tumors," "Glial Cell Tumor," "Tumor, Glial Cell," "Tumors, Glial Cell," "Mixed Glioma," "Glioma, Mixed," "Gliomas, Mixed," "Mixed Gliomas," "Malignant Glioma," "Glioma, Malignant," "Gliomas, Malignant," "Malignant Gliomas," AND "BRAF" AND "targeted therapy," "combination therapy," "dual therapy." |
| Web of Science | "dabrafenib," "GSK 2118436," "GSK2118436," "GSK-2118436," AND "trametinib," "JTP 74057," "JTP74057," "JTP-74057," "GSK 1120212," "GSK1120212," "GSK-1120212," AND "BRAF-mutant glioma," "pediatric glioma," "BRAF V600E mutation," "Glioma," "Gliomas," "Glial Cell Tumors," "Glial Cell Tumor," "Tumor, Glial Cell," "Tumors, Glial Cell," "Mixed Glioma," "Glioma, Mixed," "Gliomas, Mixed," "Mixed Gliomas," "Malignant Glioma," "Glioma, Malignant," "Gliomas, Malignant," "Malignant Gliomas," AND "BRAF" AND "targeted therapy," "combination therapy," "dual therapy." |
| Google Scholar | "dabrafenib," "GSK 2118436," "GSK2118436," "GSK-2118436," AND "trametinib," "JTP 74057," "JTP74057," "JTP-74057," "GSK 1120212," "GSK1120212," "GSK-1120212," AND "BRAF-mutant glioma," "pediatric glioma," "BRAF V600E mutation," "Glioma," "Gliomas," "Glial Cell Tumors," "Glial Cell Tumor," "Tumor, Glial Cell," "Tumors, Glial Cell," "Mixed Glioma," "Glioma, Mixed," "Gliomas, Mixed," "Mixed Gliomas," "Malignant Glioma," "Glioma, Malignant," "Gliomas, Malignant," "Malignant Gliomas," AND "BRAF" AND "targeted therapy," "combination therapy," "dual therapy." |
| Embase | "dabrafenib," "GSK 2118436," "GSK2118436," "GSK-2118436," AND "trametinib," "JTP 74057," "JTP74057," "JTP-74057," "GSK 1120212," "GSK1120212," "GSK-1120212," AND "BRAF-mutant glioma," "pediatric glioma," "BRAF V600E mutation," "Glioma," "Gliomas," "Glial Cell Tumors," "Glial Cell Tumor," "Tumor, Glial Cell," "Tumors, Glial Cell," "Mixed Glioma," "Glioma, Mixed," "Gliomas, Mixed," "Mixed Gliomas," "Malignant Glioma," "Glioma, Malignant," "Gliomas, Malignant," "Malignant Gliomas," AND "BRAF" AND "targeted therapy," "combination therapy," "dual therapy." |
